# Unveiling the Interplay Between the Human Microbiome and Gastric Cancer: A Review of the Complex Relationships and Therapeutic Avenues

**DOI:** 10.3390/cancers17020226

**Published:** 2025-01-12

**Authors:** Jenan Al-Matouq, Hawra Al-Ghafli, Noura N. Alibrahim, Nida Alsaffar, Zaheda Radwan, Mohammad Daud Ali

**Affiliations:** 1 Department of Medical Laboratory Sciences, Mohammed Al-Mana College for Medical Sciences, Al Safa, Dammam 34222, Saudi Arabia; h.alghafli@machs.edu.sa (H.A.-G.); n.alibrahim@machs.edu.sa (N.N.A.); n.alsaffar@machs.edu.sa (N.A.); zahedaa@machs.edu.sa (Z.R.); 2 Department of Pharmacy, Mohammed Al-Mana College for Medical Sciences, Al Safa, Dammam 34222, Saudi Arabia; mohammadd@machs.edu.sa

**Keywords:** microbiome, gastric cancer, microbiome modulation, chemotherapy, immune checkpoint blockade

## Abstract

The human microbiota plays a crucial role in maintaining overall health and well-being. The gut microbiota has been implicated in developing and progressing various diseases, including cancer. This review highlights the related mechanisms and the compositions that influence cancer pathogenesis with a highlight on gastric cancer. We provide a comprehensive overview of the mechanisms by which the microbiome influences cancer development, progression, and response to treatment, with a focus on identifying potential biomarkers for early detection, prevention strategies, and novel therapeutic interventions that leverage microbiome modulation. This comprehensive review can guide future research and clinical practices in understanding and harnessing the microbiome to optimize gastric cancer therapies.

## Simple Summary

The gastric microbiome has shown an association between its diversity and gastric cancer development. We point out the contributing biomarkers of the stomach microbiome and their pathogenic contribution to tumor development, aiming to highlight the opportunity of using the microbiome for early detection and raising the possibility of modulating the microbiome for therapeutic purposes.

## 1. Introduction

Human microbiota, a complex community of microorganisms that inhabit various sites of the human body, has surfaced as a necessary component in maintaining overall health and well-being. The gastrointestinal tract (GIT) is one of the key internal sites known for its rich microbial communities, often referred to as the gut microbiota. The normal gut microbiota is involved in several normal functions of the body, such as the fermentation of carbohydrates to produce energy-source short-chain fatty acids (SCFAs), synthesis of vitamins, stimulation of immune response, nutrient absorption, and the detoxification of toxins by generating bactericidal agents [1,2].

The human microbiome exhibits remarkable diversity, varying significantly between individuals and across different body sites. Genetics, age, diet, lifestyle, and environmental exposures collectively shape such unique microbial composition. Immediately after birth, the neonatal gut microbiome is established and shaped by maternal microbiota, duration of gestation, and an individual’s genetic makeup. The microbiome is later moderated by lactation, environmental exposures, and dietary intake [3]. Fat, protein, and fiber significantly influence the composition of the gut microbiota and the production of metabolic byproducts. For instance, A fiber-rich diet has been shown to enhance the abundance of beneficial bacteria, such as *Bifidobacteria* and *Lactobacilli*, which produce SCFAs. In contrast, a fat-rich diet may increase the abundance of pathogenic bacteria, such as *Bacteroides* and *Firmicutes* [4]. Further changes in the diet and immune system occurring at older age can furthermore affect the gut microbial composition [5]. The spatial distribution of these bacterial communities varies significantly along the GIT, with distinct populations residing in the stomach, small intestine, and large intestine, each adapted to their specific microenvironment and functions [6].

There is a bidirectional interaction between the host and the gut microbiota, where the gut microbiota not only influences the host’s health but also the host can regulate the diversity of the microbiota and their metabolites [7]. The host engages several mechanisms to maintain the gut microbiota’s composition and metabolite profiles. These mechanisms involve pH, mucus, and antimicrobial peptides secreted by intestinal epithelial cells which play significant roles in eliminating pathogenic microorganisms [8]. Additionally, immune cells recognize and eliminate pathogenic bacteria while developing tolerance towards beneficial commensal bacteria [9]. The immune system contributes to the balance of microbiota composition through the secretion of antimicrobial peptides and immunoglobulin A (IgA). Research has demonstrated that the binding of IgA to specific bacteria in the intestine inhibits their overgrowth and encourages microbial diversity [10]. However, under dysbiosis conditions, characterized by an imbalance between beneficial and harmful microbiota, the function of secretory IgA (sIgA) may be compromised, resulting in unregulated bacterial proliferation [11]. On the other hand, the gut microbiota plays pivotal roles in regulating various physiological processes and maintaining homeostasis of the host. Microbiota-derived products, such as SCFAs, stimulate the proliferation and differentiation of epithelial cells, thereby reinforcing epithelial barrier integrity [12]. Beneficial bacteria also promote the production of antimicrobial peptides and mucus by intestinal epithelial cells, establishing physical and chemical barriers against pathogen colonization [13].

Dysbiosis can significantly influence the body’s immune responses and may contribute to the pathogenesis of various diseases, including several types of cancer, thereby highlighting the potential of the microbiome as a critical player in both cancer development and treatment [14,15,16]. Tumorigenesis linked to the microbiome has been increasingly studied. An association has been found in gastrointestinal cancers, underlining both the tumor suppression and oncogenic effects of the microbiome [17,18]. Specific microbial species have been identified as risk factors for certain cancers. For example, the presence of *Fusobacterium nucleatum* in colorectal tissue has been associated with adverse patient outcomes, suggesting that targeting the gut microbiome through dietary interventions, probiotics, and antibiotics may offer a promising strategy for cancer prevention and treatment [14,19]. The interplay between the microbiome and various host factors, such as genetic predisposition and environmental influences, underscores the complexity of this relationship, necessitating further investigation to fully elucidate how microbial variations may serve as biomarkers for cancer risk and treatment. Therefore, understanding the mechanisms by which microbiota influences genetic and immune modulation can unveil novel therapeutic avenues, particularly in the context of enhancing the efficacy of the existing cancer treatments or developing microbiome-targeted therapies that specifically aim to restore microbial balance and function in cancer patients and potentially alleviate the cancer risks associated with dysbiosis. This review will highlight the complex interactions between specific microbial compositions and gastrointestinal cancer pathogenesis. Through understanding these interactions, we aim to summarize potential biomarkers for gastric cancer (GC) that may aid in early detection, prevention strategies, and novel therapeutic interventions that leverage microbiome modulation to guide future research and clinical practices.

## 2. Mechanisms of Microbiome-Induced Cancer Development

**Figure 1:**
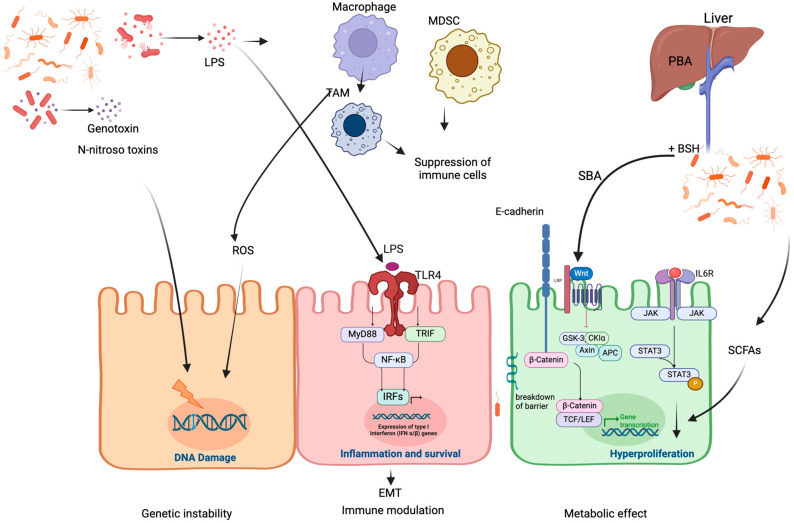
Mechanisms of the microbiome-induced cancer development seen in the gut.

Gut microbiota dysbiosis and its implication in cancer pathogenesis are increasingly being studied. We summarize the main mechanisms that have been unraveled for this contribution, in gastrointestinal cancer under genetic instability, induction of cancer-promoting inflammation and immunity, and metabolic effects (Figure 1).

### 2.1. Microbiota-Induced Genetic Instability

One of the carcinogenic effects exerted by microbiota is the direct or indirect induction of genetic instability and malignant transformations. Several studies have demonstrated that the gut microbiota induces damage to host cells’ DNA, resulting in the accumulation of mutations in their genome. These mutations, along with altered DNA repair response, can alter the expression of key oncogenes and/or tumor suppressor genes which ultimately results in malignant transformations and oncogenesis [20,21,22]. The gut microbiota-induced DNA damage occurs by either the secretion of genotoxins such as (N-nitroso compounds), activation of immune responses, and the accumulation of reactive oxygen species (ROS) or reactive nitrogen intermediates (RNIs) [20]. For instance, *Escherichia coli* was shown to be involved in the development of a unique mutational signature in colorectal cancer (CRC) through the secretion of colibactin. This genotoxin induces DNA double-strand breaks through alkylating adenines [21]. The production of superoxide by macrophages following *Enterococcus faecalis* infection was also found to cause DNA damage in epithelial cells and contribute to CRC development [23,24]. Moreover, the gut microbiota can induce DNA damage by interfering with normal DNA damage response activation. An example of such interference is the inhibition of p53 tumor suppressor activity by the gut microbiota-derived metabolite gallic acid. This was found to cause host DNA damage response dysfunction and the accumulation of malignant transforming mutations [22].

### 2.2. Microbiota-Induced Immune Modulation

Another mechanism through which microbiota can promote cancer development is through immune modulation during chronic inflammation. Chronic inflammation is a key player in all carcinogenesis stages and several of the gastrointestinal (GI) malignancies were shown to be preceded by chronic inflammations. CRC, for example, is predisposed by chronic inflammatory bowel diseases. Also, gastroesophageal reflux disease (GERD) or Barret’s esophagus are risk factors for esophageal adenocarcinoma. Microbiota-induced immune activation can stimulate multiple alterations in the cells and their surrounding microenvironment which promote tumorigenesis [20]. The gut microbiota might manipulate host immunity through enhancing pro-tumorigenic T-cell activation. A study demonstrated that during pancreatic cancer progression, tumor-associated microbiota enhanced pro-tumorigenic CD4 T cell activation, which resulted in the production of pro-inflammatory and pro-tumorigenic factors [25]. Similarly, enterotoxigenic species like *Bacteroides fragilis*, *Peptostreptococcus anaerobic*, and *F. nucleatum* were demonstrated to be implicated in CRC development through the induction of T helper 17 (Th17), MDSCs function modulation, TAMs recruitment, and reduced NK cytotoxicity [26]. Moreover, microbiota-produced SCFAs were shown to promote intestinal immunity through the triggering of CD4^+^- and innate lymphoid cell (ILC)-mediated IL-22 production by the G-protein receptor 41 (GPR41) [27].

Cancer development can also occur through immune evasion, which is usually achieved by either the inhibition of NK cells and other T cells or the recruitment of myeloid-derived suppressor cells (MDSCs) and tumor-associated macrophages (TAMs). *F. nucleatum* was found to be involved in bacterium-dependent, tumor-immune evasion by directly inhibiting the cytotoxicity of natural killer cells as well as the activity of other T cells. This inhibition was achieved through the binding of *F. nucleatum* fibroblast activation protein 2 (Fap2) to the human T cell immunoglobulin and ITIM domain (TIGIT) inhibitory receptors on the immune cells [28].

### 2.3. Microbiota-Induced Metabolic Effects

Abnormal shifts in microbiota composition or localization are associated with different metabolic effects that lead to increased epithelial barrier permeability or the translocation of bacteria and bacterial products to other organs. During initial colonization, the dysbiosis of microbiota composition can elevate harmful microbiota carcinogenic metabolites [20,24]. Heavily involved carcinogenic metabolites produced by the gut microbiota are the secondary bile acids deoxycholic acid (DCA), Lithocholic acid (LCA), and taurodeoxycholic acid (TDCA). These metabolites are produced by the deconjugation of amino acid residues from hepatocyte primary bile acids by bacterial salt hydrolases (BSHs) in the small intestine [29]. These metabolites are then used by the gut microbiota as nutrients to support their colonization and bile resistance. The secondary bile acid DCA was found to promote colorectal cancer through the induction of the Wnt signaling pathway, which enhances tumor proliferation and survival. It was also found to interfere with the negative regulation of epithelial cell proliferation by suppressing the activity of the intestinal nuclear hormone receptor Farnesoid X receptors (FXRs), in which reduced activity was implicated in the development of cancers [30].

Also, LCA was shown to induce the STAT-3-dependent elevation of the chemokine interleukin-8, which has been frequently detected in serum samples of CRC and is thought to be associated with poor prognosis [31,32]. Moreover, the secondary bile salt TDCA was found to strongly correlate with *Prevotella melaninogenica*, a lipopolysaccharide (LPS)-producing bacteria in the gastric juices of gastric cancer patients. This was found to be linked to the activation of the IL-6/JAK1/STAT3 pathway, which increases proliferative potential.

Additionally, SCFAs are metabolites that may promote carcinogenesis despite their anticarcinogenic effects and their ability to improve gastrointestinal immunity [33]. The most abundant forms of SCFAs are acetate, propionate, and butyrate. SCFAs are believed to exert tumor-suppressing activities including anti-inflammatory, apoptosis, and autophagy at normal physiological levels [34]. Butyrate, for example, was shown to inhibit colon cancer progression through the reduction in cell viability and apoptosis [35]. Other studies have also documented a decreased level of SCFAs in the gut due to a lower abundance of butyrate-producing bacteria in the gut, namely Firmicutes and Bacteroides, and an increase in pathogenic bacteria [36]. The drop in these beneficial metabolites weakens the intestinal lining and can negatively impact the growth of epithelial cells, as well as cell differentiation and the expansion of regulatory T-cells (Treg-cells) which control immune response.

However, SCFAs were shown to exert pro-tumorigenic actions in gastrointestinal carcinogenesis if their levels increase to exceed the host’s tolerance. It was shown that in dysbiosis background, butyrate was found to support tumor-promoting microenvironment and inflammation [37]. Butyrate was also shown to be associated with the promotion of colon cell proliferation, survival, and growth by acting as an energy source [38].

Enterotoxigenic *Bacteroides fragilis ETBF* produces an enterotoxin with metalloprotease-like enzymes termed Bacteroides fragilis toxin (BFT) [39]. This toxin plays a pivotal role in cleaving the extracellular domain of E-cadherins, which is crucial for cell adhesion and maintaining intestinal barrier integrity [40]. A compromised intestinal barrier allows toxins and bacteria to penetrate the gut lining, and such increased gut permeability contributes to the pathogenic “Leaky Gut” phenomenon [36]. Additionally, several in vitro studies described the tumor-promoting effect of BFT on colonic epithelial cells, outlined by the activation of the β-catenin signaling pathway which leads to (I) increased secretions of inflammatory interleukins such as IL8 and (II) increased c-yc translations and cellular proliferation [41,42]. Hence, the microbial presentence of EPTB by itself acts as a potent factor that alters gastrointestinal epithelial structure and physiology, which in turn further facilitates oncogenic transformation and chronic inflammation.

## 3. Microbiota and Gastric Cancer

For many years, it was believed that there is no existence of a microbiota in the stomach due to its acidic environment, mucus layer thickness, and the effect of the antimicrobial agent—nitric oxide—produced by the gastric juice [43]. *Helicobacter pylori* (*H. pylori*), a spiral-shaped bacterium, was then discovered to colonize and damage the gastric mucosa by various virulence factors. These factors include cytotoxins and ureases, which convert urea to ammonia neutralizing the acid environment, and adhesin proteins, which can help avoid immune destruction through attaching the bacteria to the cells that line the inner surface of the gastric mucus layer. Infection with *H. pylori* activates a cascade of histologic changes that progress from chronic superficial gastritis to atrophic gastritis, intestinal metaplasia, and dysplasia, before developing to GC [44]. *H. pylori* has been well recognized for playing a role in oncogenesis via three mechanisms: (I) the activation of tumor signaling pathways by its exerted cytotoxins VacA and CagA into the host cells, (II) the production of ROS and its consequent activation of inflammatory pathways, and (III) its ability to change the gastric acidity by produced ammonia [45]. Single-nucleotide polymorphisms (SNPs) in several *H. pylori* virulence genes may contribute to carcinogenesis. A study has demonstrated an SNP in the 171L-type HtrA allele correlated with the disruption of the adherens cellular junctions, causing increased CagA injection which in turn interferes with various oncogenic signaling pathways and provokes malignant changes [46].

The estimated global prevalence of *H pylori* infection has decreased over the years, however, estimated to be 43.1% (40.3–45.9) in 2022 [47]. Although *H. pylori* infection is strongly associated with GC, only 1–3% of the infected individuals develop GC. This raises the question of whether other microbiomes can influence the progression to gastric cancer.

Changes in the stomach environment allow other microbiota to colonize the stomach [43]. Over a thousand clones of microbiota have been identified in the stomach with 65% of the strains also found in the human mouth. These acid-resistant transient bacteria can be present in the stomach arriving from the bacteria of the oral cavity or reflux from the duodenum [48]. Therefore, H. *pylori* acts as a carcinogen due to its resulting inflammation and gastric environment changes. Consequently, the colonization of transient microorganisms coming from the oral cavity or intestine can increase, resulting in fluctuations in stomach microbiota richness and diversity which may temper the immune responses and further progress *H. pylori*-induced inflammation and dysplastic alterations of the gastric mucosa. Nonetheless, the exact relationship between *H. pylori* and other microbiota is still controversial. Studies revealed that patients with *H. Pylori* infection had a lack of *Bacteroidetes* and *Proteobacteria* phyla and a relative abundance of *Streptococcus* and *Prevotella* genera [49]. Other studies reported a higher abundance of *Proteobacteria*, *Spirochetes*, and *Acidobacteria* and a decreased abundance of *Actinobacteria*, *Bacteroidetes*, and *Firmicutes* in *H. pylori*-positive patients [50]. This necessitates the importance of the identification of gastric microbiome composition and the changes associated with carcinogenesis in an attempt to predict early diagnosis and treatment.

## 4. Microbiome as a Predictive Biomarker for Gastric Cancer

Research has recently focused on the stomach microbiomes’ contribution to gastric carcinogenesis. Analysis of the gastric microbiome using culture-based methods or next-generation sequencing demonstrated alteration in the microbial diversity and domination of several bacteria genera to be associated with gastric cancer (Table 1). One example is Lactobacillus, which is known to carry anti-inflammatory effects and relieve gastrointestinal conditions [51] and may contribute to carcinogenesis when imbalanced. This was demonstrated by the production of ROS, DNA damage, and the reduction of nitrate to nitrite, which in turn can activate oncogenesis, enhance angiogenesis, and inhibit apoptosis [52,53,54]. Nitrite is a product of reduction by nitrate reductase, an enzyme which is found to be produced from oral bacterium including *Veillonella*, *Streptococcus*, *Staphylococcus*, *Haemohilus*, *Campylobacter*, *Pasteurella*, *Neisseria*, *Rothia*, *Actinomyces*, and *Prevotella* [55,56]. Studies have demonstrated the association of nitrite production in gastric cancer with the increase in the microbiota Bacteroidetes, Lactobacillus, and Firmicutes [52,57]. Nitrite in turn produces carcinogenic nitrosamine in acidic conditions of the stomach [58] and can lead to the generation of DNA double-strand breaks within the host’s epithelial cells [59]. Additionally, *Fusobacterium* has been shown to promote gastric carcinogenesis via FadA adhesin, activating the β-catenin signaling pathway and various inflammatory properties of the cells [60].

## 5. The Impact of the Microbiome on Cancer Treatment

Due to studies into the molecular mechanisms of cancer genesis, progression, and metastasis, several cancer treatments have been invented, including hormone therapy, targeted therapy, immunocheckpoint blockage (ICB), radiation therapy, chemotherapy, and surgery.

Chemotherapy continues to represent the mainstay of cancer treatment since it prevents cancerous cells from growing and spreading throughout the body. This therapy method improves the quality of life for cancer patients by either eliminating cancer cells or reducing symptoms. The drugs used for chemotherapy disrupt the formation of DNA, replication, and division of cells by interfering with the different phases of the cell cycle. This mechanism primarily targets quickly growing cancer cells, but it also impacts healthy normal cells in the body, leading to a number of negative consequences [83].

Finding a possible function of the microbiota of the gut as a biomarker for forecasting the effectiveness of cancer treatment is of interest to clinical researchers. The possible role of the gut microbiota as a predictive factor in cancer treatment has been demonstrated by numerous research studies [81,82,84]. The effects of the gut microbiome and locally resident bacteria on GIT cancer and the treatment of cancer are covered separately here.

### 5.1. Microbiota Affects the Efficacy of Chemotherapy

Although different cytotoxic drugs have various mechanisms, they exhibit antitumor efficacy via targeting cell division and DNA integrity in highly proliferative cancer cells [85]. Around 40 drugs are metabolized by gut microbes, including methotrexate (MTX) and irinotecan (IRT), indicating that the gut microbiota can contribute to the response to chemotherapeutics [86]. Evidence demonstrated a reduced therapeutic effect of oxaliplatin and 5-fluorouracil (5-FU) in response to an antibiotic cocktail in colorectal cancer [87,88]. Notably, ROS induced by commensal bacteria-dependent inflammatory response plays a crucial role in maintaining the cytotoxicity of oxaliplatin [87]. In contrast, some bacteria could promote chemotherapy resistance. For instance, *F. nucleatum* was associated with post-chemotherapy recurrence and decreased efficacy of oxaliplatin and 5-FU in colorectal cancer patients [89]. Mechanistically, *F. nucleatum* could induce autophagy activation to promote chemoresistance in a TLR4/MYD88-dependent manner [90].

Moreover, *F. nucleatum* may increase BIRC3, which directly blocks the caspase cascade that results in 5-FU resistance [89]. Intriguingly, fecal microbiota transplant (FMT) from healthy wild-type donor mice could restore the gut microbiota composition and downregulate TLRs and MYD88 after chemotherapy [91], offering a promising strategy to remodel intestinal microecology and overcome chemoresistance in colorectal cancer.

Platinum-based chemotherapy drugs are widely used in cancer treatment, and they exert a cytotoxic effect by binding to DNA, thereby impairing DNA replication. It was found that Gram-positive antibiotics decrease the efficacy of cisplatin in a clinical setting [92]. Another standard chemotherapeutic is Gemcitabine (GEM), which is an antimetabolite, a chemotherapy drug that interferes with the DNA or RNA inside the cancer cells to stop them from growing or dividing. It is applied to pancreatic cancer, non-small cell lung cancer, breast cancer, etc. [93]. The antitumor action of GEM depends on its activation or degradation, where cytidine deaminase plays a pivotal role in its degradation process [93]. Growing evidence suggests that bacteria can metabolize GEM into an inactivated form by deamination. It was demonstrated that *Gammaproteobacteria* and *Mycoplasma hyorhinis* could promote GEM resistance in pancreatic and breast cancers [94,95]. Furthermore, the chemotherapy response of breast cancer patients was enriched with the *Clostridiales*, *Bifidobacteriaceae*, *Turicibacteraceae*, and *Prevotellaceae* gut microbiota [85]. Bawaneh et al. also found that a high abundance of *Akkermansia muciniphila* was favorable for enhancing the doxorubicin (DOX) efficacy in breast cancer [96].

After capecitabine plus oxaliplatin treatment, several changes were observed in the gut microbiota, such as increased pathogenic bacteria and decreased probiotics, including *Dorea, Streptococcus,* and *Roseburia* [86]. Moreover, the abundance of Firmicutes was shown to be significantly reduced after chemotherapy [86,97]. Sougiannis et al. speculated that *Firmicutes* could affect the efficacy of chemotherapy through its capability to produce SCFAs [97]. Interactions between microbes and cancer treatments raise the possibility of changing the microbiome to improve treatment efficacy and reduce side effects as listed in Table 2.

### 5.2. Gut Microbiota Reduces Chemotherapy-Induced Toxicity

Gastrointestinal (GI) toxicity, a dose-limited side effect of many chemotherapeutics, affects approximately 80% of cancer patients [98]. This toxicity, mainly referring to mucositis, can lead to severe complications like bacteremia and sepsis. This not only necessitates chemotherapy dose reduction and treatment cessation but also poses a significant economic burden for cancer patients. The current lack of adequate medical measures to prevent and treat chemotherapy-induced gastrointestinal toxicity underscores the urgent need for solutions in this area.

According to clinical guidelines, IRT (irinotecan) is the first-line chemotherapy for advanced colorectal cancer patients. Nevertheless, the primary limitation of IRT is its associated complication of diarrhea, which may result in significant dehydration, electrolyte imbalance, and nutritional deficiencies [99,100]. The key to reducing the side effects of IRT is to decrease the concentration of its active form (SN38) in the GIT [99]. Selenium-enriched *Bifidobacterium longum*, a probiotic strain, could be a promising therapeutic agent for IRT-induced diarrhea. This strain has been shown to reduce the pro-inflammatory cytokines IL-1β and IL-18 and upregulate the expression of tight-junction proteins occludin and ZO-1, thereby improving the integrity of the intestinal barrier and reducing the severity of diarrhea [101]. In addition to intestinal toxicity, the gut microbiota is associated with chemotherapy-induced nerve injury. Peripheral neuropathic pain is often caused by paclitaxel (PTX) or platinum compounds. Neurons and glial cells are susceptible to chemotherapy-triggered inflammatory factors that are well-known starters of nociceptive pain in neuropathy [102]. By boosting the expression of opioid and cannabinoid receptors in the spinal cord, decreasing nerve fiber damage in the paws, and modifying the serum pro-inflammatory cytokine concentration, oral probiotic treatment in mice can prevent PTX-induced neuropathy [103]. Furthermore, a randomized controlled trial reported that probiotic supplements could mitigate chemotherapy-related cognitive impairment by attenuating synapse injury, oxidative stress, and glial activation in the central nervous system [104]. Evidence that the gut microbiota is associated with chemotherapeutics’ efficacy and adverse effects is summarized in Table 3.

While antibiotics are frequently used to prevent chemotherapy-associated infections, it is crucial to be cautious about their potential side effects. Given the pivotal role of the gut microbiota, using antibiotics for infection could lead to other complications, such as neuropathic pain or diarrhea [107]. This underscores the need for a thorough assessment of the pros and cons of antibiotics and the consideration of treatment strategies that specifically target gut microbes. The list of active clinical research assessing the connection between changes in the gut microbiota and the incidence of late toxicities in cancer survivors is compiled in Table 2.

### 5.3. Role of the Microbiome in Gastric Cancer Immunotherapy

ICB has been the most studied strategy of cancer immunotherapy. To stop tumor cells from eluding the immune system, it outlines how to precisely target ICB, such as cytotoxic T lymphocyte-associated protein (CTLA-4) and programmed cell death-1 (PD-1)/PD-ligand [108]. Additionally, by over-stimulating the immune system of the patient, ICB can cause immunological-related adverse events, including moderate to severe and even fatal GI, endocrine, and dermatological side effects [109]. Optimizing patient results, which are still difficult to achieve in terms of evaluation; planning for treatment; and therapy monitoring requires a customized strategy [110]. Research also reveals that there is an association of gut microbes with immunotherapy efficacy [111]. Microbiome-targeting strategies, such as probiotics, prebiotics, and fecal microbiota transplantation, have been studied to improve immunotherapy response [112].

Certain gut bacterial ecosystems can influence the effectiveness of immunotherapy according to preclinical research on mice models. Preclinical studies on mouse models suggest that the efficacy of immunotherapy may be influenced by gut bacterial communities. Mice with good gut flora, for instance, responded more strongly to CTLA-4 inhibition than mice treated with antibiotics or kept in germ-free environments [113]. Certain bacteria, including *Burkholderia cepacian*, have shown susceptibility to immunotherapy when combined with *Bacteroides thetaiotaomicron* [113]. Additionally, research has revealed that specific bacterial strains, such as *Lactobacillus johnsonii*, *Bifidobacterium pseudopodium*, and species from the genus *Olsenella*, isolated from tumors treated with ICIs, can significantly enhance the effectiveness of ICIs in animal models [114]. Studies have also indicated that *Bifidobacterium* strains improve the efficacy of anti-PD-1 immunotherapy by promoting tumor infiltration and boosting immune cell activity [115,116]. Moreover, isolated strains like *Lactobacillus paracasei* sh2020 and *Lactobacillus kefiranofaciens* ZW18 have shown potential in enhancing the effectiveness of anti-PD-1 treatment [116].

The relationship between the composition of the microbiome and the results of immunotherapy in cancer patients has been the subject of numerous clinical research as described in Table 4. *Prevotella*, *Ruminococcaceae*, and *Lachnospiraceae* were significantly more abundant in a particular subgroup of immune checkpoint blockade responders. Shotgun metagenomics analysis, meanwhile, showed that the response to anti-PD-1/PD-L1 therapy is positively correlated with the bacteria in the gut that may create SCFAs, such as *Streptococcus*, *Lactobacillus*, and *Eubacterium*, across a variety of gastric cancer types [117].

## 6. Positive Impact of Gastric Microbiome on Cancer Treatment Through Immune Modulation

### 6.1. Immune Enhancement

Microorganisms have an intriguing role in the battle against cancer. They enhance the immune system and directly destroy tumor cells. Certain microorganisms can stimulate the immune system to recognize and attack cancer cells more effectively. This approach forms the basis of cancer immunotherapy and involves two main mechanisms. Firstly, the Activation of Innate Immunity: Microbial components like lipopolysaccharides (LPS) or peptidoglycans can activate Toll-like receptors (TLRs) on immune cells, prompting the release of cytokines and enhancing the immune response [132]. One example is Bacillus Calmette-Guérin (BCG), a live attenuated strain of *Mycobacterium bovis*, used in bladder cancer immunotherapy. It triggers a local immune response, leading to the destruction of cancer cells [133]. The second mechanism is Stimulating Adaptive Immunity: Engineered bacteria or viruses can deliver tumor antigens to antigen-presenting cells (APCs), enhancing the activation of T cells targeting cancer cells. One example is Oncolytic viruses, such as the genetically modified herpes simplex virus (*Talimogene laherparepvec* or T-VEC), which can infect and lyse tumor cells while stimulating an anti-tumor immune response [134].

### 6.2. Direct Tumor Cell Toxicity

Microorganisms and their metabolites can exhibit selective toxicity to tumor cells through apoptosis induction, cell cycle arrest, or metabolic disruption. Bacterial toxins like hemolysins or exotoxins produced by *Clostridium* or *Listeria* species can directly target hypoxic tumor environments, releasing toxins that kill cancer cells [135]. Additionally, natural products derived from microorganisms have been the source of many chemotherapeutic agents. For instance, *Streptomyces* produce doxorubicin and actinomycin D, effective anticancer drugs [136]. Also, *Aspergillus terreus* produces lovastatin, which can inhibit cancer cell proliferation by targeting cholesterol biosynthesis pathways essential for tumor growth [137].

### 6.3. Oncolytic Microorganisms (Bacteria and Virus)

Oncolytic bacteria and viruses are engineered or naturally occurring organisms that specifically infect and kill cancer cells while sparing normal tissues. *Salmonella typhimurium* and *Clostridium* spp. have been engineered to target hypoxic tumor environments. These bacteria can disrupt tumor blood vessels, making them more susceptible to immune attack [138]. Oncolytic viruses like adenoviruses or reoviruses are used to replicate selectively and lyse cancer cells. These viruses can also serve as delivery systems for therapeutic genes [139].

### 6.4. Synergistic Effects with Conventional Therapies

Microorganisms can complement traditional cancer therapies, improving their efficacy or reducing their side effects. Certain probiotics can reduce the gastrointestinal toxicity of chemoradiation, maintaining the patient’s gut microbiota and immune balance. Studies show that microbial therapies can improve the outcomes of checkpoint inhibitors like anti-PD-1 or anti-CTLA-4 by reshaping the tumor microenvironment [140].

## 7. Microbiota Modulation Approaches

### 7.1. Antibiotics and Microbiome Disruption

According to a recent study, using oral antibiotics raises the risk of colon cancer [141]. The microbiome can also determine clinically significant symptoms of gastrointestinal toxicity, which could offer a fresh justification for when and how to target the microbiome to lessen the acute and long-term problems brought on by the disturbance of the gastrointestinal milieu [142]. The application of this new understanding should concentrate on creating novel microbial strategies to hasten mucosal repair and maintaining and enhancing the gut microbiota prior to chemotherapy. Consequently, limiting the extent and length of mucosal damage may avoid stomach toxicity’s subsequent effects [142].

### 7.2. Dietary Interventions

An imbalanced diet may induce carcinogenesis, as it can alter the gut microbiota by skewing the abundance of specific species and metabolites [143]. Ascorbic acid treatment after *H. pylori* eradication was shown to dramatically reduce the intestinal metaplasia of the gastric mucosa in a prospective randomized trial [144]. Chinese interventional studies on the role of high dosages of folic acid in gastric carcinogenesis in beagles have shown that folic acid plays an important role as a chemopreventive agent for gastric cancer [145]. Although many trials have shown promising outcomes, several research studies have been unsuccessful in showing the benefits of the dietary chemoprevention of gastric cancer. A Colombian randomized control research study on its high-risk group found that vitamin C and β-carotene had no significant effects as chemopreventive medications for stomach cancer [146].

Furthermore, a different study found no impact on the risk of stomach cancer from supplementing with a combination of vitamin C, vitamin E, and selenium [147]. Thus, additional pharmacokinetic research is needed to ascertain the therapeutic chemoprevention of different dietary supplements.

### 7.3. Probiotics, Prebiotics, and Synbiotics

Live bacteria and yeast, also referred to as probiotics, are primarily found in fermented foods like kefir, yogurt, and other supplements [148]. On the other hand, prebiotics are nondigestible food elements, such as onions, whole grains, garlic, and asparagus, which help to promote the growth and activity of gut bacteria. Last but not least, postbiotics are the metabolic byproducts that are normally created by probiotic bacteria in the gut, such as peptides, specific bacterial cell components, and the SCFA butyrate. Currently, the role of probiotics in enhancing chemotherapy efficacy is gaining an increasing amount of attention. Three putative food-derived probiotics (*L. plantarum* S2, *L. pentosus* S3, and *L. rhamnosus* 14E4) showed an increased effect in colorectal cancer cells by releasing butyrate, exopolysaccharides, and other peculiar proteins [149]. There is a perception that probiotics must face acidic gastric juice, diverse digestive enzymes, and bile salts when going through the GIT. Changes in live microorganisms to unknown compounds may lead to the loss of some of these beneficial functions [150,151]. Prebiotics—including inulin, fructooligosaccharide (FOS), and galactooligosaccharides (GOS)—are fibers promoting the growth of specific groups of anaerobic colonic indigenous bacteria; they are undigestible by endogenous enzymes in the small intestine but are actively fermented by colonic bacteria, selectively promoting the growth of beneficial bacteria [152]. Notably, although the importance of the gut microbiota in mediating chemotherapy response has been emphasized, each bacterial species’ specific role still needs more investigation. In addition to being utilized by gut microbes to produce beneficial metabolites, prebiotics are deemed as a crucial component of some specific delivery systems to maintain stability, enhance efficacy, and reduce side effects of chemotherapeutics. For example, inulin and DOX conjugate were developed to improve cancer therapy with increased cytotoxicity, which could be attributed to more robust binding to DNA, more accessible access into cells, and larger molecular size to prevent efflux pumps from removing the conjugate from tumor cells [153]. To date, although scientific interest in the role of prebiotics during cancer is growing, original studies remain lacking.

Nevertheless, the protective effect against cancer seems to be due to intrinsic anti-inflammatory and proapoptotic properties rather than a modulatory effect on microbiota [154,155]. The impact of inulin consumption, a natural dietary soluble fiber consisting of a mixture of oligo- and polysaccharides, should be studied given its prebiotic potential, technological properties, and beneficial effects on the gut microbiota [156]. Moreover, several randomized controlled trials demonstrated a potential role for synbiotics (a combination of prebiotics and probiotics) in reducing adverse events in patients receiving neoadjuvant chemotherapy; however, it has not been investigated in potentiating chemotherapy efficacy. The administration of synbiotics could lower the ratio of neutropenia, lymphopenia, diarrhea, and bacteremia in esophageal cancer patients during neoadjuvant chemotherapy [153,157]. Besides its synergistic effect, future research may focus on developing newer or personalized combinations of synbiotics to target various cancer types with different gut microbiota changes.

The interplay of prebiotics, probiotics, and postbiotics is crucial for effective microbiome modulation. By combining both prebiotics and probiotics, it is possible to create a synergistic effect that enhances microbial diversity in the gut and restores microbial balance. In addition, fecal microbiota transplantation (FMT) is another strategy of microbial composition modulation. FMT involves the transfer of stool from a healthy donor into the GIT of a patient. The purpose is to restore a balanced and healthy gut microbiome, which is essential for proper digestion, immune function, and combating the growth of cancerous cells [148,158].

#### 7.3.1. Microbiome Modulation Attempts in Cancer

Microbiome modulation has emerged as a feasible alternative or complementary treatment strategy for several cancer types. It has been proven potentially effective in treating different types of cancers such as colon, gastric, pancreatic, liver, breast, lung, prostate, and ovarian cancers. Here, we shed light on some of the anticancer microbiome modulation attempts.

In vitro experiments have examined the impact of probiotics such as Pediococcus pentosaceus and Lacticaseibacillus paracasei, on the suppression of colon cancer proliferation [159,160]. Administrating strains that belong to the Lacticaseibacillus genus in models with colon cancer also demonstrated reduced tumor growth [123]. Additionally, Lacticaseibacillus bacteria have been tested in colorectal cancer patients for 12 weeks and demonstrated improved bowel movements [161]. Probiotic metabolites of these bacteria can also contribute to the suppression of cancer growth. For instance, treating HT-29 cells with the cell-free supernatant of Lacticaseibacillus rhamnosus not only prevented metastasis and proliferation but also promoted their apoptosis [127]. Another study documented the inhibition of colon cancer growth when treated with probiotic extracellular polysaccharides (EPSs) and S-layer protein (Slp) [162]. The dietary uptake of inulin inhibited tumor growth via the activation of both CD8 + T and CD4 + T cells, these cells play an important role in inducing anti-tumor immune responses [163]. SCFAs inhibit the growth of pathogenic bacteria by lowering the PH and regulating inflammation and tumor growth [164]. Similarly, beneficial metabolites can exert their effects through several mechanisms, including enhancing the gut barrier function, modulating the immune response, and restoring the balance of gut microbiota composition. By balancing the microbiome, probiotics can inhibit the growth of pathogenic bacteria and reduce inflammation, both of which are linked to colorectal carcinogenesis. Furthermore, treating HT-29 with a mixture of Lactobacillus sporogenes, Clostridium butyricum, and Saccharomyces cerevisiae (either as heat-activated or live strains) was associated with high apoptosis and suppressive effect on tumor cells [165]. Also, postbiotics of heat-killed *Levilactobacillus brevis* and *Lacticaseibacillus paracasei* enhanced the expression of Bax, caspase-3, and caspase-9 and inhibited the expression of Bcl-2 on HT-29 cells [166].

Similarly, probiotic-tumor inhibition was demonstrated through an anti-inflammatory effect in colon cancer. The utilization of Lactobacillus bacterial strains (i.e., *Lactobacillus acidophilus*, *Lactobacillus lactis*, *Lactobacillus casei*) and Bifidobacterium bacterial strains (i.e., *Bifidobacterium longum*, *Bifidobacterium bifidum*, and *Bifidobacterium infantis*) significantly reduced pro-inflammatory cytokines such as TNF-α, IL-6, IL-10, IL-12, IL-17A, and IL-17C [167,168]. In line with this notion, a study has shown that administering the metabolites of *Bifidobacteria* in fermented milk stimulates the production of IL-10, an anti-inflammatory cytokine, while reducing the release of IL-8, a pro-inflammatory cytokine, in epithelial cells [168]. FMT has also been tested in vitro on mice and showed a reduction in immunosuppressive cells, exemplified by Foxp3+ Treg cells, and an increase in CD8+ T and CD49b+ NK [169], all of which highlights the potential therapeutic role of probiotics, prebiotics, postbiotics, and FMT on colon cancer treatment.

In gastric cancer patients, Zheng et al. studied the effect of a mixture of *Bifidobacterium infantis*, *Enterococcus faecalis*, *Lactobacillus acidophilus*, and *Bacillus cereus* on patients who underwent partial gastrectomy. The study showed a reduction in harmful bacteria of the genus *Streptococcus* and an enrichment of beneficial bacteria [170]. The utilization of *lactobacillus reuteri* in vitro resulted in downregulating the Urokinase plasminogen activator, which plays a vital role in gastric cancer progression [171]. Furthermore, increased uptake of raffinose, as a prebiotic, seemingly reduces the risk of gastric cancer formation [172].

#### 7.3.2. Potential Limitations of Microbiome Modulation Using Probiotics

While probiotics hold significant potential for treating cancer and various other diseases, several clinical trials documented harmful metabolic activities due to the intake of probiotics strains represented by gas, bloating, D-lactic acidosis, bowel ischemia, or diarrhea, particularly during the first phases of the treatment [173,174]. More concerning, is the risk of developing opportunistic infections due to the uptake of probiotic supplementations among immunocompromised individuals such as those undergoing chemotherapy, organ dysfunction or organ failure, and HIV patients [175,176,177]. These opportunistic infections range in clinical severity from mild to systematic and severe infections leading to bacteremia, endocarditis, or sepsis, and the infection itself could stem directly from the commercial strains available in probiotic supplementations or from an endogenous strain that flares due to the manipulation of microbial compositions [175,176,177]. For instance, two cases of bacteremia were reported due to the uptake of probiotic strains of the genus *Lacticaseibacillus* [178]. Another study reported sepsis due to probiotic uptake in a patient who underwent an aortic valve replacement [179]. These insights emphasize a concern regarding what would an “optimum dosage and duration of treatment” of probiotics look like for each patient in clinical settings, and the need for well-developed, customized, and evidence-based ‘safety guidelines’ to ensure the effective and successful practice of treatment.

### 7.4. Engineered Microbes in Cancer Treatment

The integration of engineered microbes into cancer treatment represents a promising frontier in oncology, leveraging the microbiome to enhance therapeutic efficacy. Facultative anaerobic bacteria can potentially colonize the necrotic center of solid tumors and the well-perfused periphery of the tumor tissue, and this gives it a therapeutic potential for cancer treatment. Since the early 2000s, utilizing the microbiome for cancer therapy has become a significant area of research [180,181]. Unlike traditional chemotherapy and radiotherapy, which primarily target peripheral tumor areas near blood vessels, engineered gut microbes such as *Salmonella*, *E. coli*, *Clostridium*, and *Bifidobacterium* can penetrate the necrotic and hypoxic regions of tumors, inducing apoptosis in cancer cells. Also, engineered microbes can produce tumor antigens fused to outer membrane vesicles in situ within the intestine after oral administration. These antigen-bearing vesicles can act as tumor vaccines, reducing tumor growth and providing protective effects against tumor re-exposure in animal models [182].

Genetically engineered strains of *Salmonella* VNP20009, which are attenuated facultative anaerobes that can penetrate the tumor because of their ability to grow in hypoxic areas, have been used to carry Sox2 shRNA construct in combination with the anti-tumor polypeptide HM-3 in a mouse xenograft model of NSCLC and enhanced the antitumor effect of HM-3 resulting in the induction of apoptosis by increasing Bax expression, cleaving caspase 3, and decreasing BCL2 [183].

The non-pathogenic probiotic *E. coli* Nissle 1917 (EcN) has been identified as a safe carrier for tumor-targeting bacterial therapy due to its probiotic nature and its ability to replicate and colonize in tumors. EcN was shown to exert a pro-apoptotic effect on colon cancer cells through up-regulation of *PTEN* and *Bax* and down-regulation of *AKT1* [184]. Invitro experiments showed genetically engineered EcN-expressing cytotoxic compounds colibactin, glidobactin, and luminmide remarkably inhibited tumor growth in mice models [185]. Moreover, EcN supernatants were shown in another study to be effective in reducing the viability of Caco-2 cancer cells and significantly reducing the activity of caspase3/7 which in turn reduced damage in intestinal epithelial cells caused by 5-fluorouracil (5-FU) chemotherapeutic drug [186]. Additionally, EcN was reprogrammed to bind to heparan sulfate proteoglycans (HSPGs) on cancer cells and secrete myrosinase, which converted dietary glucosinolates into sulforaphane. The produced sulforaphane inhibited cancer cell growth and promoted apoptosis, leading to colorectal tumor clearance. This combined approach led to a nearly complete cancer cell inhibition in vitro [187].

Another study demonstrated bioengineering EcN for therapeutic applications to release the cytokine Granulocyte-macrophage colony-stimulating factor (GM-CSF) and produce checkpoint inhibitors by blocking nanobodies against PD-L1 and CTLA-4 at neoplastic sites. This enhanced the T-cell infiltration in the adenoma and resulted in a 50% reduction in adenoma burden in a mouse CRC model [188]. Another study demonstrated engineered EcN to enhance the synthesize of butyrate from glucose. When administered to human colorectal cancer cells, the biobutyrate induced G1 phase cell cycle arrest and activated a p53-independent mitochondrial apoptosis pathway [189].

Furthermore, administering an engineered strain of *Enterococcus hirae* in which its prophage expresses a major histocompatibility complex (MHC) epitope enhanced the response to immunotherapy, and immune checkpoint inhibitors such as anti-programmed cell death 1 ligand 1 (anti-PD-1) antibody treatment, these therapies can reactivate T cells, allowing them to attack cancer cells more effectively due to cross-reactivity between tumor antigens and the bacteriophage [190]. Another study showed that the presence of the essential amino acid; L-arginine in tumors is crucial for a robust anti-tumor T-cell response and raising their levels in tumors could significantly enhance the effectiveness of immune checkpoint inhibitors, such as PD-L1-blocking antibodies [191]. (EcN) were, therefore, bioengineered to convert an accumulated metabolite waste; ammonia to L-arginine in tumors. This bacterial colonization led to increased intra-tumoral L-arginine levels, a higher number of tumor-infiltrating T cells, and a significant synergistic effect with PD-L1-blocking antibodies in tumor clearance [192].

*Clostridium perfringens* (*C. perfringens*) enterotoxin (CPE) has emerged as an effective cancer therapy due to its selective cytotoxicity [193]. CPE targets the transmembrane tight junction proteins claudin-3 and claudin-4 receptors, which are overexpressed in several cancers including colon carcinoma. CPE binding to these proteins induces pore formation of the cell membrane, leading to rapid cell death [194]. A study used a translation-optimized CPE-expressing vector (optCPE) and tested it in colon cancer and melanoma. The study demonstrated that optCPE gene transfer is a promising strategy for targeted claudin-3- and -4-overexpressing colon carcinomas, resulting in cellular membrane permeability and generating a calcium influx, triggering rapid apoptosis [194]. Collectively, these findings demonstrate that engineered microbial therapies can effectively modify the tumor microenvironment, which can be used to enhance the efficacy of immunotherapies, and the underlying mechanisms of engineered microbes, such as their ability to target specific tumor microenvironments, can be adapted for different cancers including gastric cancer.

**Figure 2:**
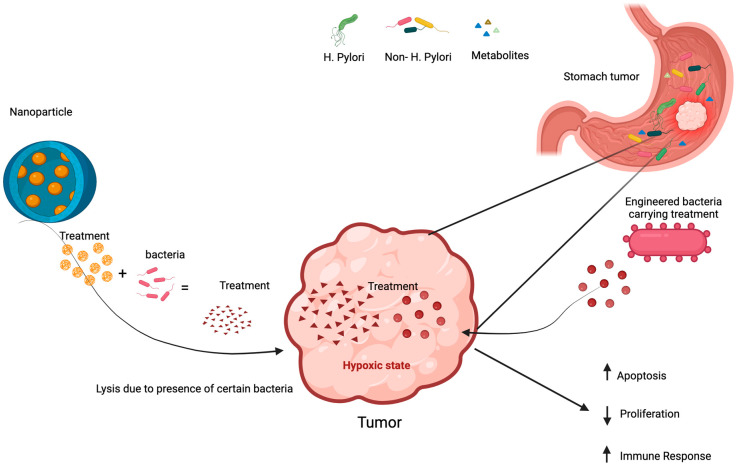
Manipulation of microbiome for potential gastric cancer treatment. (↑ = Increase, ↓ = Decrease).

Gastric cancer presents unique challenges, including its complex tumor microenvironment and the presence of various microbial communities in the stomach. Engineered microbes could potentially target gastric tumors by delivering therapeutic agents directly to tumor cells or by modulating the immune response. For instance, strains like *Salmonella* VNP20009, known for their ability to reach hypoxic tumor regions, could be modified to deliver specific genes or proteins that induce apoptosis in gastric cancer cells. Furthermore, *ECN* could be engineered to enhance local immune responses or produce metabolites that inhibit tumor growth (Figure 2). Further exploration of microbial engineered therapies in gastric cancer could lead to innovative treatment modalities that improve patient outcomes. Research into these applications is necessary to fully understand the potential and efficacy of engineered microbes in combating gastric cancer.

### 7.5. Nanoparticles Technology in Microbiome Manipulation

Nanotechnology has emerged as a promising approach for cancer therapy, particularly through its potential to modulate the microbiome [195,196]. A study has demonstrated a lipid–protamine–DNA nanoparticle gene delivery system encapsulated in a plasmid designed to produce an LPS-binding fusion protein (LPS-trap) [197]. Since LPS is an important product of the gut Gram-negative microbiota, associated with intestinal inflammation and CRC progression through the TLR4- NF-kB pathway [198], the delivery of these LPS-trap nanoparticles to the orthotopic tumors has led to the blockage of LPS-TLR4 interactions in the tumor microenvironment. This treatment resulted in increased T-cell infiltration and significantly reduced tumor growth compared to anti-PD-L1 therapy alone. The combination of nanotechnology with anti-PD-L1 therapy further enhanced tumor burden reduction by over fivefold compared to LPS-trap treatment alone [197,198].

Another recent study has demonstrated how the metabolites secreted by tumor-associated bacteria (TAB) can catalyze the degradation of doxorubicin-loaded nanoparticles for on-demand drug release at tumor sites. Specifically, a triple-layered nanogel composed of polyethylene glycol, poly (ε-caprolactone)/polyphosphoester nanogel (TLND) was engineered to degrade in the presence of bacterial lipase, releasing encapsulated doxorubicin into the tumor microenvironment. In vitro experiments confirmed that doxorubicin release triggered cytotoxicity in hepatoma cells and occurred only in the presence of bacterial lipase [90,199]. In a hepatoma xenograft model infected with lipase-secreting *Staphylococcus aureus*, the systemic administration of TLND reduced tumor weight by more than twofold compared to standard doxorubicin treatment, and over fourfold compared to TLND treatment without TAB [199].

These findings highlight the potential for designing nanotechnologies that respond to specific bacterial metabolites within the tumor microenvironment, paving the way for a new treatment modality that enables the stimuli-responsive and on-demand release of anticancer drugs mediated by bacterial metabolism and by modulating microbiome-tumor interactions (Figure 2).

## 8. Conclusions

Different types of cancers are associated with a unique microbial signature, highlighting the potential role of the microbiome in cancer development, progression, and treatment. Gastric cancer microbiome signatures could eventually serve as biomarkers for early cancer detection, help in personalizing cancer treatment, and improve patient outcomes through modulating the microbiome. However, more research is needed to fully understand these relationships and how they can be harnessed for clinical benefit. Furthermore, the composition of an individual’s gastric microbiome can be influenced by variable factors such as diet, genetics, age, and environment. These intraindividual variations along with the potential side effects of probiotics make it difficult to predict who will benefit from probiotic supplementation and further complicate the development of one-size-fits-all probiotic solutions.

Despite these advances, clinically viable products derived from microbes for cancer diagnosis and therapy remain limited. One major challenge is the capability of engineered microbes to detect and target specific tumor regions accurately. From a therapeutic standpoint, ensuring that the molecules produced by these engineered microbes can cross the gastric barrier and reach distant tumor sites poses another significant obstacle, especially for complex drugs like paclitaxel and vinblastine. The effective biosynthesis of such drugs necessitates intricate genetic engineering. Given the high genetic and phenotypic variability among cancer patients, a one-size-fits-all approach for engineered microorganisms is impractical, even within individual patients. Thus, any gastric microbiome engineering strategy, whether used in combination with or sequentially alongside other treatments, must be applied in the context of personalized medicine. Nevertheless, advancements in using engineered microbes for colorectal cancer can potentially be adapted for gastric cancer treatment. Nonetheless, gastric cancer presents unique challenges, including a complex tumor microenvironment and diverse microbial communities, engineered microbes could be tailored to deliver therapeutic agents or modulate immune responses in gastric tumors, potentially improving patient outcomes.

In conclusion, while promising progress has been made in utilizing engineered microbes and nanotechnology for cancer treatment, further research is essential to refine these approaches, optimize their efficacy, and explore their potential applications in gastric cancer. The dynamic nature of cancer necessitates personalized strategies that account for individual patient variability, ultimately aiming to improve therapeutic outcomes in oncology.

## Figures and Tables

**Table 1:** Commonly identified microbiota associated with gastric cancer and how they contribute to carcinogenesis. All the microbiota listed have been identified on a DNA level.

Cancer-Promoting Microbe (Genus Level)	Phylum	Ref.	Pathogenesis/Mechanism Linked to Gastric Cancer	Ref.
*Helicobacter pylori*	Proteobacteria	[57,61,62,63]	Convert Urea to ammonia through urease and neutralizing the acidity of the stomach. Excrete toxins VacA which induces apoptosis and CagA which induces pro-inflammatory cytokines and inflammation.	[45]
*Escherichia*/*Shigella*	*Proteobacteria*	[61,64,65]	Produce N-nitroso toxins generate DNA double-strand breaks within the host’s epithelial cells, thus promoting a transient cell cycle arrest, allowing for genomic mutations to arise, and finally leading to tumor formation.	[66]
*Campylobacter*	Proteobacteria	[63,67]	Produce N-nitroso toxins.	[56]
*Enterococcus*	Firmicutes	[61,64]	Produce reactive oxygen species (ROS) and reduced expression of the MMR gene involved in DNA repair.	[68]
*Staphylococcus*	Firmicutes	[69]	Produce N-nitroso toxins.	[70]
*Streptococcus*	Firmicutes	[48,57,63,71,72,73]	Produce N-nitroso toxins. Produce Short-chain fatty acids (SCFAs)—Acetic acid.	[63]
*Lactobacillus*	Firmicutes	[57,61,67,72,73]	Produce N-nitroso compounds. Produce Short-chain fatty acids (SCFAs).	[52,70]
*Megasphaera*	Firmicutes	[72,74]	---	
*Veillonella*	Firmicutes	[48,57,63,67,71,72]	Produce N-nitroso toxins.	[56]
*Clostridium*	Firmicutes	[75]	Produce N-nitroso toxins.	[75]
*Peptostreptococcus*	Firmicutes	[63]	Induces the activation of the PI3K–Akt pathway, leading to increased cell proliferation and (NF-κB) activation, and triggers a pro-inflammatory response.	[76]
*Lactococcus*	Firmicutes	[54]	Lactic acid-producing bacteria produce reactive oxygen species (ROS), which induce DNA damage, reduce nitrate to nitrite, drive the activation of oncogenes, enhance angiogenesis, and inhibit apoptosis.	[54]
*Parvimonas*	Firmicutes	[63]	Enhanced secretion of inflammatory cytokines l-23 and Il-17.	[77]
*Bulleidia*	Firmicutes	[73]	---	
*Haemophilus*	Pseudomonadota	[48,67,71,73]	Produce N-nitroso toxins.	[56]
*Pasturellaceae*	Pseudomonadota	[48,71]	Produce N-nitroso toxins.	[56]
*Neisseria*	Pseudomonadota	[63,73]	Produce N-nitroso toxins.	[56]
*Leptotrichia*	Fusobacteriota	[67]	Produce endotoxins which activate pro-inflammatory interleukins (IL)-1β, IL-6, IL-8, and IL-10.	[78]
*Fusobacterium*	Fusobacteriota	[57,65,67,72]	FadA adhesin, which binds to E-cadherin, activates β-catenin signaling and accordingly, various inflammatory pathways.	[60,79]
*Actinomyces*	Actinobacteria	[74]	Produce N-nitroso toxins.	[56]
*Rothia*	Actinobacteria	[48,71]	Produce N-nitroso toxins.	[55]
*Bacteroides*	Bacteroidetes	[65,74]	---	[80]
*Prevotella*	Bacteroidetes	[61,72,73]	Produce N-nitroso toxins. Produce Short-chain fatty acids (SCFAs)—Acetic acid.	[56]
*Nitrospirae*	Nitrospirota	[81]	Produce N-nitroso toxins.	[56]
*Dialister*	Bacillota	[82]	---	
*Granulicatella*	Bacillota	[82]	---	
*Herbaspirillum*	Pseudomonadota	[82]	---	
*Comamonas*	Pseudomonadota	[82]	---	
*Chryseobacterium*	Bacteroidota	[82]	---	

**Table 2:** Current list of clinical trials with microbiome- and cancer treatment-induced short- and long-term side effects (source: ClinicalTrials.gov).

NCT Number	Study Type	Types of Cancer	Purpose	Patients (n) (Adult)	Intervention Model Description	Study Status
NCT06088940	An interventional double-blinded, placebo-controlled, randomized study; parallel assignment	Cancer	To compare the effects of probiotics versus placebo on gut bacteria and their correlation with gastrointestinal and psychosocial functions.	66	Survivors of cancer will receive one probiotic (strains of *Lactobacillus* and *Bifidobacterium*) and one placebo (maltodextrin) capsule every day for 12 weeks. The effect of probiotics on diarrhea/gas/bloating/anxiety/fatigue symptoms and cognitive function will be observed.	Not yet recruiting
NCT06022822	Interventional, randomized placebo-controlled trial	Prostate cancer	This phase 2 randomized control trial assesses the effect of Uro-A supplementation compared to placebo in men with biopsy-confirmed prostate cancer undergoing radical prostatectomy progressive disease.	90	The primary endpoint will be analyzed using a linear regression model.	Recruiting or phase 2
NCT05349227	Crossover assignment in an open-label, interventional randomized research	Breast cancer, lung cancer, stomach cancer, ovarian cancer, and lung cancer	To assess the gut microbiota in fecal samples at the beginning of the trial and six months after enrollment, as well as to track changes in depression, cognitive performance or impairment, and sleep-related impairment.	625	Individuals will be assigned to groups that will be monitored by wrist-worn devices and receive either six months of digital coaching right after or six months of monitoring and digital health coaching thereafter.	Recruiting
NCT04700527	A Randomized Controlled Study	Cancer	To assess and compare gastrointestinal toxicity from radiation therapy between subjects who receive therapeutic short-chain fatty acid and those who receive placebo, identifying a safe, low-cost therapeutic to reduce gastrointestinal toxicity from therapeutic or environmental radiation.	122	GI toxicities (PRO-CTCAE v5 for patients and CTCAE v5 for physicians) will be recorded and compared between the 2 groups to identify any differences.	Recruiting/phase 2
NCT06039644	Parallel Assignment, Randomized Controlled Trial	Breast Cancer	To explore after consumption of probiotics of lactobacillus composite strain powder sachets for 6 months in BC chemotherapy, and whether it assists patients in alleviating the side effects of chemotherapy.	100	The questionnaire will be finished to record within 24 weeks the side effects, including nausea, vomiting, diarrhea, stomatitis, peripheral neuropathy, skin rashes, and hand-food syndrome before and after the treatment.	Recruiting
NCT04775355	An observational prospective study	Prostate cancer	To examine the gut microbial population after radiation and androgen deprivation therapy and identify alterations linked to post-treatment toxicity.	30	Questionnaires will be filled out by participants prior to, during, and following radiation treatment.	Recruiting
NCT05112614	An observational prospective study	Cancer	This study examines how gut microbiome can affect cancer therapy in patients with cancer undergoing cancer therapy or stem cell transplantation. Information from this study may help medical professionals improve the way cancer treatment condition is delivered and increase its efficacy and success.	5000	Will be performed using the Shogun pipeline for metagenomics data followed by analysis using QIIME 2.0.	Recruiting

**Table 3:** Effects of gut microbiota on cancer treatment.

Gut Microbiome	Experimental Model	Chemotherapy Regimen	Types of Cancer	Effects		Mechanism	Ref.
				Efficacy	Toxicity		
*Fusobacterium nucleatum*	Mice	Oxaliplatin and 5-fluorouracil	Colorectal cancer	↓	-	Inducing autophagy activation to promote chemoresistance in a TLR4/MYD88-dependent manner; upregulating BIRC3 that directly inhibited caspase cascade	[90]
*Mycoplasma hyorhinis*	Mice	Gemcitabine	Breast cancer	↓	-	Metabolizing GEM into inactive form by deamination	[95]
*Akkermansia muciniphila Lactobacillus*	Mice	Doxorubicin	Breast cancer	↑	-	-	[96]
*Bifidobacterium longum*	Mice	Irinotecan	Irinotecan-induced diarrhea	-	↓	Decreasing the pro-inflammatory cytokines IL-1b and IL-18	[102]
Probiotic Mixture Slab51^®^	Mice Stool sample	Paclitaxel	Paclitaxel-induced neuropathy	-	↓	Modulating the serum pro-inflammatory cytokine concentration	[104]
*Lactobacillus plantarum*	Bacterial Cell culture		Colorectal cancer	↑	-	Secreting metabolites to increase the expression of the butyrate transporter	[105]
*Bacteroides vulgatus*	Mice	5-fluorouracil	Colorectal cancer	↑	-	Decreasing the abundance of F. nucleatum, and more efficient capacity of DNA repair	[106]

↑ = Increase, ↓ = Decrease, - = No Effect.

**Table 4:** Human gut microbes enriched in responders to immunotherapy.

Cancer Type	Immunotherapy	Microbes Enriched in Responders	Sequencing Methods	Patients (n)	Ref.
Gastrointestinal	anti-PD-1/PD-L1	*Prevotella/Bacteroides ratio*, *Prevotellaceae*, *Ruminococcaceae*, *Lachnospiraceae, Eubacterium*, *Lactobacillus*, and *Streptococcus*	16S rRNA (V3-V4) and metagenomics	74 adults	[118]
Hepatobiliary	anti-PD-1	*Lachnospiraceae bacterium* GAM79, Alistipes sp. Marseille-P5997, *Ruminococcus calidus*, and *Erysipelotichaceae bacterium* GAM147	metagenomic	65 adults	[119]
HCC	anti-PD-1	*Faecalibacterium*, *Blautia*, *Lachnospiracea incertae Sedis*, *Megamonas*, *Ruminococcus*, *Coprococcus*, *Dorea*, and *Haemophilus*	16S rRNA (V3-V4)	35 adults	[120]
HCC	nivoluma	Akkermansia	16S rDNA (V3–V4)	11	[121,122]
HCC	camrelizumab	*Akkermansia muciniphila* and *Ruminococcaceae* spp.	16S rDNA (V3–V4)	11	[121,122]
Thoracic carcinoma	anti-PD-1	Akkermansiaceae, Enterococcaceae, Enterobacteriaceae, *Carnobacteriaceae*, and *Clostridiales* Family XI	16S sRNA (V4)	42	[123,124]
B cell lymphoma	anti-CD19 CAR-T	*Bacteroides*, *Ruminococcus*, *Eubacterium*, and *Akkerman*	metagenomic	172	[125]
Pan-cancer	immune checkpoint inhibitors	*Trichophyton benhamiae*, *Cryptococcus amylolentus*, *Suillus clintonianus*, *Pseudogymnoascus* sp. 05NY08, *Schizosaccharomyces octosporus*, *Podospora anserina*, and *Verticillium longisporum*	metagenomic	862	[126,127]
Prostate Cancer	pembrolizumab	*Streptococcus*	16S rRNA and qPCR	23	[128]
NSCLC	anti-PD-1/PD-L1	*Desulfovibrio*, *Bifidobacterium*, *Anaerostipes*, *Faecalibacterium*, and *Alistipes*	16S rRNA (V3-V4)	75	[129]
NSCLC	immune checkpoint inhibitors	*Phascolarctobacterium*	16S rRNA (V3-V4)	69	[130,131]

## Data Availability

The current study did not produce or analyze any datasets.
